# Author Correction: One-step Conjugation of Glycyrrhetinic Acid to Cationic Polymers for High-performance Gene Delivery to Cultured Liver Cell

**DOI:** 10.1038/s41598-023-44255-8

**Published:** 2023-10-18

**Authors:** Yue Cong, Bingyang Shi, Yiqing Lu, Shihui Wen, Roger Chung, Dayong Jin

**Affiliations:** 1 Institute of Pharmacy, Pharmaceutical College, Henan University, Jin Ming Avenue, Kaifeng, 475004 Henan China; 2 College of Life Sciences, Henan University, Jin Ming Avenue, Kaifeng, 475004 Henan China; 3 Advanced Cytometry Labs, ARC Centre of Excellence for Nanoscale BioPhotonics (CNBP), Macquarie University, Sydney, NSW 2109 Australia; 4 Faculty of Medicine & Health Sciences, Macquarie University, Sydney, NSW 2109 Australia

Correction to: *Scientific Reports* 10.1038/srep21891, published online 23 February 2016

This Article contains errors.

As a result of errors during figure assembly, incorrect data is displayed in the following panels:

- Figure 6C PPI (G4) with CHO, both images;
- Figure 6C GA-PPI-4 with CHO, both images;
- Figure 6C PPI(G4) with HepG2, both images;
- Figure 6C GA-PPI-5 with HepG2, upper image;
- Figure S8A 24 h after gene transfection image;
- Figure S10 PPI(G4) N/P 10, left image;
- Figure S10 GA-PPI-4 N/P 10, left image.

The corrected Figure 6C, Figure S8A and Figure S10 appear below, as Figure 1, 2, and 3.

**Figure 1:**
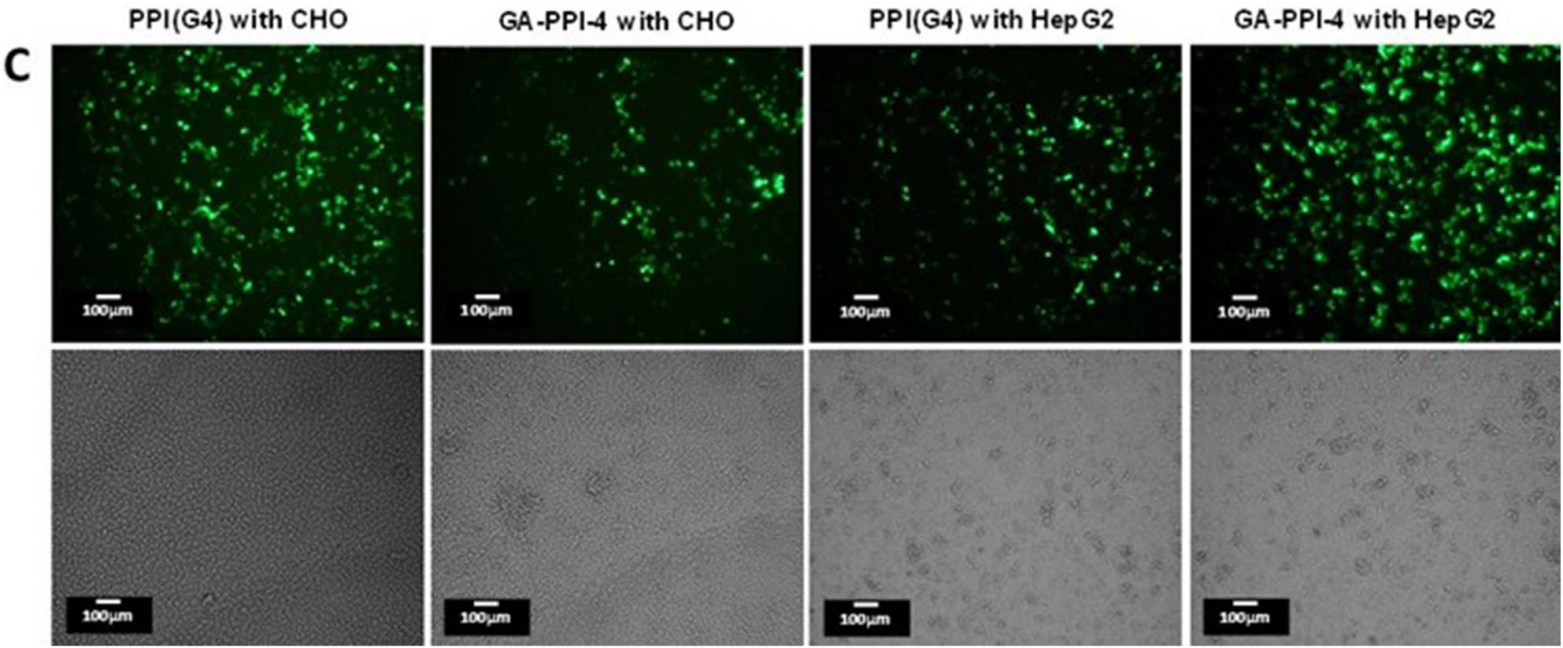
The corrected panel 6C.

**Figure 2:**
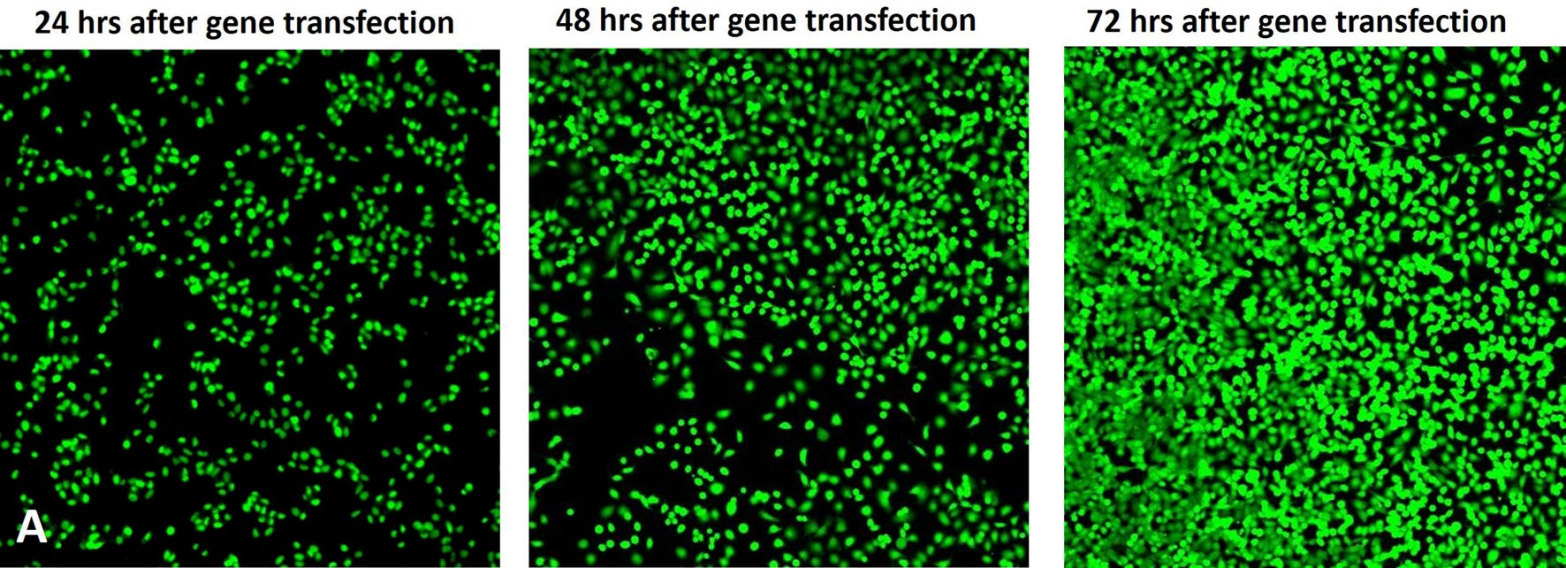
The corrected panel S8A.

**Figure 3:**
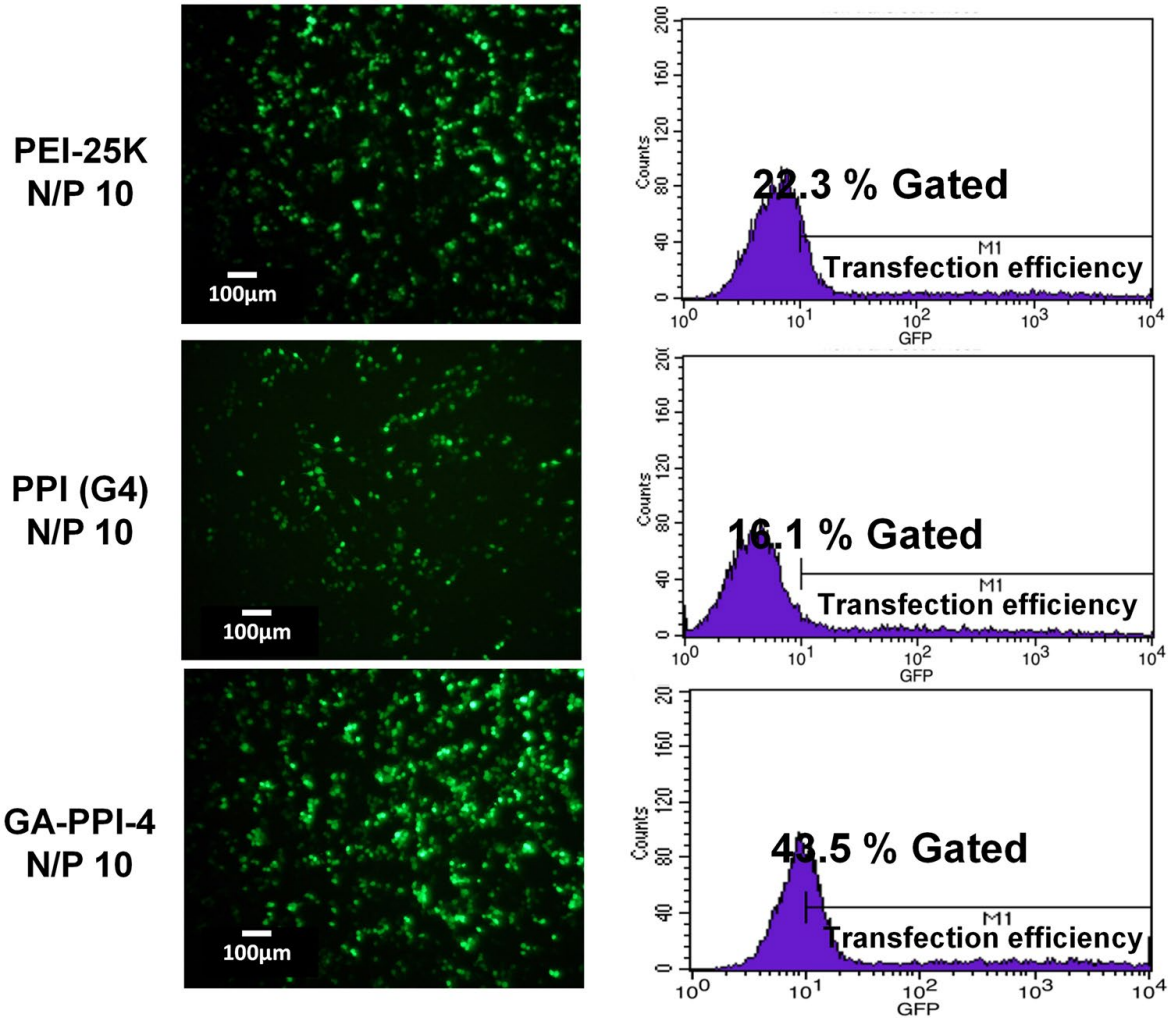
The corrected Figure S10.

